# Annual transcriptome of a key zooplankton species, the copepod *Calanus finmarchicus*

**DOI:** 10.1002/ece3.8605

**Published:** 2022-02-22

**Authors:** Laura Payton, Céline Noirot, Kim S. Last, Jordan Grigor, Lukas Hüppe, David V. P. Conway, Mona Dannemeyer, Amandine Suin, Bettina Meyer

**Affiliations:** ^1^ 11233 Institute for Chemistry and Biology of the Marine Environment Carl von Ossietzky University of Oldenburg Oldenburg Germany; ^2^ 84597 Section Polar Biological Oceanography Alfred Wegener Institute Helmholtz Centre for Polar and Marine Research Bremerhaven Germany; ^3^ Plateforme bio‐informatique GenoToul MIAT INRAE UR875 Mathématiques et Informatique Appliquées Toulouse Castanet‐Tolosan France; ^4^ 1292 Scottish Association for Marine Science Oban UK; ^5^ Neurobiology and Genetics Theodor‐Boveri Institute Biocentre University of Würzburg Würzburg Germany; ^6^ Helmholtz Institute for Functional Marine Biodiversity (HIFMB) University of Oldenburg Oldenburg Germany; ^7^ Marine Biological Association of the UK Plymouth UK; ^8^ Plateforme Génomique INRAE US 1426 GeT‐PlaGe Centre INRAE de Toulouse Occitanie Castanet‐Tolosan France

**Keywords:** annual rhythms, copepods, diapause, transcriptome, wildlife, zooplankton

## Abstract

The copepod *Calanus finmarchicus* (Crustacea, Copepoda) is a key zooplanktonic species with a crucial position in the North Atlantic food web and significant contributor to ocean carbon flux. Like many other high latitude animals, it has evolved a programmed arrested development called diapause to cope with long periods of limited food supply, while growth and reproduction are timed to take advantage of seasonal peaks in primary production. However, anthropogenic warming is inducing changes in the expected timing of phytoplankton blooms, suggesting phenological mismatches with negative consequences for the N. Atlantic ecosystem. While diapause mechanisms are mainly studied in terrestrial arthropods, specifically on laboratory model species, such as the fruit fly *Drosophila*, the molecular investigations of annual rhythms in wild marine species remain fragmentary. Here we performed a rigorous year‐long monthly sampling campaign of *C*. *finmarchicus* in a Scottish Loch (UK; 56.45°N, 5.18°W) to generate an annual transcriptome. The mRNA of 36 samples (monthly triplicate of 25 individuals) have been deeply sequenced with an average depth of 137 ± 4 million reads (mean ± SE) per sample, aligned to the reference transcriptome, and filtered. We detail the quality assessment of the datasets and provide a high‐quality resource for the investigation of wild annual transcriptomic rhythms (35,357 components) in a key diapausing zooplanktonic species.

## Graphical abstract

While diapause mechanisms are mainly studied in terrestrial arthropods, specifically on laboratory model species, such as the fruit fly *Drosophila*, the molecular investigations of annual rhythms in wild marine species remain fragmentary. Here we performed a rigorous year‐long monthly sampling campaign of *C. finmarchicus*, a key zooplanktonic species, in a Scottish Loch (UK; 56.45°N, 5.18°W) and generated an annual transcriptome.

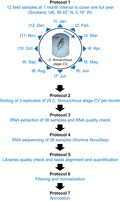

## 1 INTRODUCTION

The calanoid copepod *Calanus finmarchicus* (Crustacea, Copepoda) is a key ecological species in the North Atlantic that often dominates zooplankton biomass between ~40° and 80°N (Helaouët & Beaugrand, 2007). Primarily herbivorous, this species has a central position in the food web as it converts sugars from micro‐algae (phytoplankton) into energy‐rich lipids that sustain higher consumers including marine fish larvae, seabirds, and whales (Beaugrand et al., 2003; Berge et al., 2012; Prokopchuk & Sentyabov, 2006). Its high abundance and primary consumer position also makes it an important contributor to ocean carbon flux (Archibald et al., 2019). Despite this, copepods are among important non‐model invertebrates for which genomic resources are still limited, principally due to their large genomes (Bron et al., 2011; Choquet et al., 2019). However, the de novo transcriptome of *C*. *finmarchicus* (Lenz et al., 2014) represents the most useful resource to date for molecular investigations in this ecologically important species.

Seasonal adaptations have evolved in zooplankton to deal with annual biotic and abiotic cycles, such as long periods of limited food resources (Baumgartner & Tarrant, 2017; Daase et al., 2021; Lenz & Roncalli, 2019). Copepods within the Calanidae and Eucalanidae families synchronize reproduction and growth with periods of high food availability, during which they accumulate large energetic reserves in sunlit surface waters (Baumgartner & Tarrant, 2017). This follows a migration of copepods into deeper water where they enter a prolonged phase of inactivity referred to as diapause, characterized by a non‐feeding state with reduced metabolism lasting 8–10 months. The energy reserves accumulated during the spring production phase sustain the animals during periods of low food supply and are crucial to fuel moulting to adult stages and reproduction in the following year. In *C*. *finmarchicus*, the seasonal cycle begins prior to, and during, the spring bloom, when the juvenile pre‐adult copepodid stage CV emerge from diapause, molt into adults, mate and spawn eggs (Baumgartner & Tarrant, 2017; Niehoff et al., 2002). The resulting offspring then develop through several nauplii and juvenile copepodid stages. The energy resource provided by the spring bloom is optimized by large lipid accumulations, specifically by the last copepodid stage CV. At this stage, CVs either directly migrate into deeper water where they enter diapause or molt into adults to produce another generation that enters diapause at a later time (Häfker et al., 2018; Johnson et al., 2008; Tarrant et al., 2014, 2016). The number of generations and timing of diapause vary according to populations and geographic locations (Häfker et al., 2018; Melle et al., 2014).

While the seasonal timing of the copepod life cycle is crucial for its survival and reproduction, broad geographic temperature increases caused by global climate change alter the seasonal dynamics of biotic and abiotic environmental cycles (Helm et al., 2013; Kharouba & Wolkovich, 2020). In particular, shifts in seasonal timing of phytoplankton blooms have been observed (Friedland et al., 2018). Concurrently, climate change induces the shift of *C*. *finmarchicus* to higher latitudes (Murphy et al., 2016; Reygondeau & Beaugrand, 2011; Saikkonen et al., 2012) with new residencies predicted in high boreal and Arctic seas (Tarling et al., 2021). Consequently, climate change may lead to phenological mismatches, where the timing of critical events between interacting species that have been finely tuned over evolutionary time scales becomes desynchronized (Helm et al., 2013; Kharouba & Wolkovich, 2020). Considering the key position of lipid‐rich copepods in the food web (Archibald et al., 2019; Beaugrand et al., 2003; Berge et al., 2012; Prokopchuk & Sentyabov, 2006), such mismatches may ultimately have consequences on energy flow pathways in ecosystems and carbon sequestration (Friedland et al., 2018). While our current understanding of the life cycle and ecology of copepods has emerged mostly from population dynamics and physiological studies (Baumgartner & Tarrant, 2017; Lenz & Roncalli, 2019), seasonal investigations on the transcriptional level are still rare and fragmentary (Clark et al., 2013; Häfker et al., 2018; Lenz, Lieberman, et al., 2021; Lenz, Roncalli, et al., 2021; Roncalli et al., 2018, 2020, 2021; Semmouri et al., 2020; Tarrant et al., 2014), as too are the processes controlling the timing of the annual life cycle (Baumgartner & Tarrant, 2017; Lenz & Roncalli, 2019).

Studying the seasonal life cycle of *C*. *finmarchicus* in its natural habitat, the open ocean, is extremely challenging. Populations are migrating vertically via diel vertical migrations (DVM) and horizontally via advection in ocean currents, and different copepod species frequently co‐occur. However, an isolated population of *C*. *finmarchicus* can be found on the west coast of Scotland in Loch Etive. In the upper basin, including Bonawe deep (~150 m), *C*. *finmarchicus* dominates the mesoplankton community (Häfker et al., 2018; Hill, 2009; Mauchline, 1987), as less than 4% of CV and CVI stage individuals have been identified as *Calanus helgolandicus* (Hill, 2009). Indeed, the Loch Etive *Calanus* species composition was analyzed in 2016 genetically, and no *C*. *helgolandicus* was detected (Choquet et al., 2017). It has been suggested that *C*. *helgolandicus* is intolerant to low salinities, such as found in Loch Etive (Helaouët & Beaugrand, 2007; Hill, 2009). Primary production in this loch is comparatively low (Wood et al., 1973), and terrestrial organic matter run‐off probably provides a supplementary food source for *C*. *finmarchicus*. Häfker et al. (2018) have described a peak of phytoplankton production in May, followed by a sharp decrease until August, and very low Chl *a* concentration from November to March. Previous studies agreed on one single synchronized cohort of *C*. *finmarchicus* each year (Clark et al., 2012, 2013; Hill, 2009; Mauchline, 1987), which would start to descend in preparation for diapause (100–150 m depth) in July, while Häfker et al. (2018) argued for a possible second generation descending to diapause depth in October. During their active phase, *C*. *finmarchicus* perform DVM, with a daytime depth ~70 m (Häfker et al., 2018). Finally, the water column at Bonawe deep is highly stratified. Salinity and temperature in the top 50 m are variable seasonally due to the fluctuations in terrestrial freshwater run‐off and tidal influence. Below 50 m, the water column is mixed with temperature and salinity extremely stable over the year (Häfker et al., 2018; Hill, 2009).

In this study, we took advantage of Loch Etive to perform a complete annual transcriptome of the copepod *C*. *finmarchicus*. The sampling campaign was conducted at Bonawe deep (UK; 56.45°N, 5.18°W) between 50 and 140 m depth, under extremely stable oceanographic conditions, but where both active and diapausing copepods are found. As copepods use multiple environmental cycles to synchronize their biological rhythms, from gene expression to behavior, the annual sampling was made at comparable phases of daily and lunar cycles (tidal and monthly cycles) (Häfker et al., 2017; Ibáñez‐Tejero et al., 2018; Last et al., 2016; Payton et al., 2021; Schmitt et al., 2011). The genetic analysis focused on the copepodite pre‐adult stage CV, which experiences all seasons and has a key role both in the active surface feeding and deep‐water diapause states. The 36 mRNA samples (3 replicates of 25 individuals per month), analyzed with a high sequencing depth, yielded a total of 4.9 billion reads, with an average of 137 ± 4 million reads (mean ± SE) per sample. In addition, we provide a quality assessment and the alignment to the reference transcriptome (Lenz et al., 2014), allowing the monthly quantification of 35,357 unique “components” in the *C*. *finmarchicus* transcriptome and the first investigation of annual transcriptomic rhythms in a key zooplanktonic species. Finally, existing annotations have been combined for further transcriptomic data exploration.

## 2 MATERIALS AND METHODS

### 2.1 Sampling design

**FIGURE 1:**
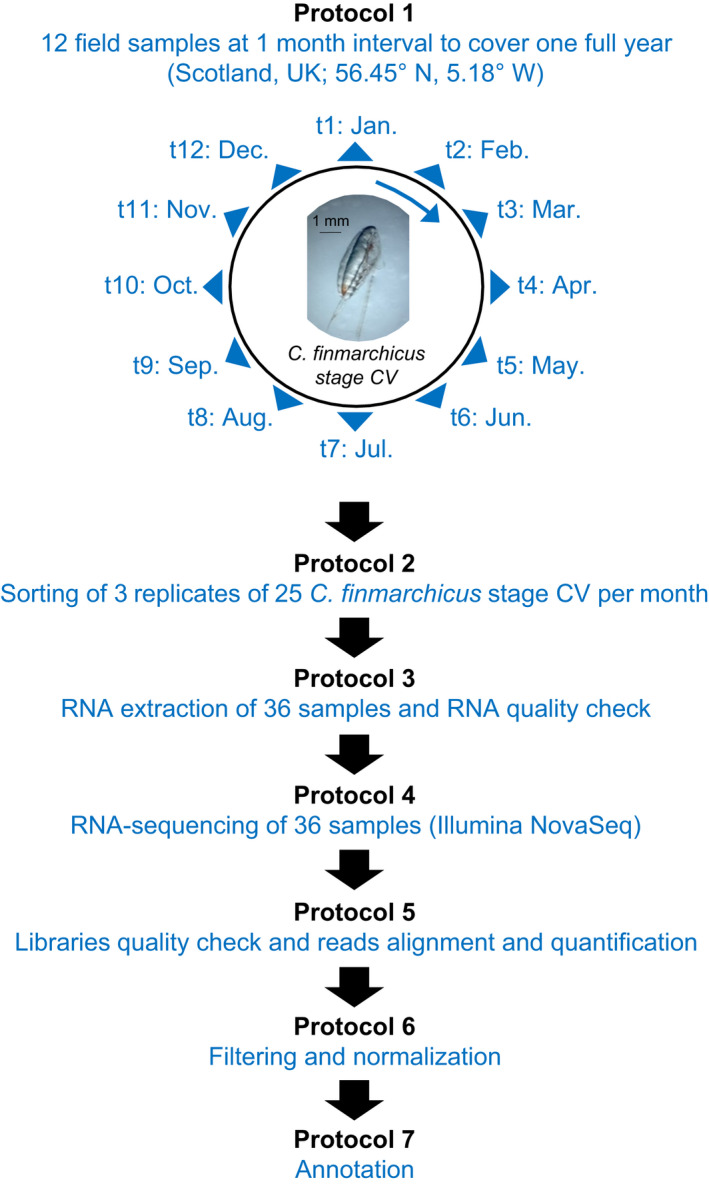
Overview of the experimental workflow used to generate the transcriptomic data output. Sample details are available in Tables S1 and S2

Sampling design and analysis strategy are presented in Figure 1, Table 1, and Table S1.

**TABLE 1:** Summary of sampling and sequencing strategy. Details are available in Tables S1 and S2

Number of time points	Sampling frequency	Total duration of sampling	Number of replicates per time point	Total number of samples	Sequencing strategy	Reads	Platform	Volume
12	1 month	1 year	3	36	RNA‐seq	Paired‐end 2 × 150 pb	Illumina NovaSeq	1 lane S4

Sampling was conducted over a year, with monthly samples from January to December 2019 (between the 1st and the 12th of each month), resulting in 12 time points. Sampling covered the winter (January–March), spring (April–June), summer (July–September), and autumn (October–December) seasons. Sampling was performed between 10:00 am and 12:15 pm, while local solar local noon is at ~12:30 pm, in order to be at a comparable phase of the daily rhythm (all times noted in UTC) (detailed in Table S2). Furthermore, sampling was performed on waxing moons (between New Moon and First Quarter Moon) and within 2:46 h of high tide, to provide comparable phases of lunar cycles (Table S2). However, while all samples were taken around high tide, some were during the flood tide and others during the ebb tide (Table S2). Copepods were collected from the RV *Calanus* (Scottish Association for Marine Science, SAMS) using a WP2‐net equipped with an opening/closing mechanism (200 μm mesh size/50 μm cod end) hauled vertically through the water column at 0.5 ms from 140 m to 50 m depth. Samples were immediately (within 5 min after retrieval) fixed as bulk samples in RNA*l* *ater* stabilization solution (Ambion). A 12 h period of incubation at 2–4˚C was allowed to soak the samples thoroughly with the stabilization solution before they were transferred to −80˚C for further transport and storage. Duplicate net sampling, with identical methodology, was carried out at the same time for species identification samples. These were fixed in 4% buffered formalin solution.

### 2.2 Site description

Sampling was conducted in Bonawe deep, the deepest point (~150 m) of Loch Etive, a sea loch on the western coast of Scotland, UK (56.45°N, 5.18°W). Two sills with a depth of ≈7 m and ≈13 m at Connel and Taynuilt villages respectively limit the exchange with the open ocean (Häfker et al., 2018). The deeper waters of the sampling station typically cycle from normoxic to hypoxic and due to the sills, overturning of these water masses happens irregularly every ≈18 months when spring tides in spring/autumn co‐occur with low precipitation (Edwards & Edelsten, 1977). Monthly CTD (conductivity, temperature, and depth) measurements were performed and archived by the British Oceanographic Data Centre (BODC) (Last & Dumont, 2021). As previously shown (Häfker et al., 2018), large seasonal changes in temperature and salinity occur above 50 m which is highly stratified, while only small seasonal changes occur in deeper waters (mixed), where net sampling was conducted. The average temperature and salinity from 140 m to 50 m depth over the year were 11.4 ± 0.3°C and 27.8 ± 0.1 psu (mean ± SE) respectively (detailed in Table S2).

### 2.3 Species identification

Formalin solution was removed from the monthly community samples by draining them through a 100 micron nylon gauze and then rinsing the sample with tap water. Animals were transferred to a round‐bottomed flask and diluted to 300 ml with tap water. After careful sample mixing, sub‐samples were taken with a 5 ml stempel pipette and transferred to a Bogorov tray. The first 200 *Calanus* were confirmed morphologically to corroborate species.

### 2.4 Copepod sorting

Molecular analysis was performed on the CV copepodite stage of *C*. *finmarchicus*, the main overwintering stage that represents the bulk of the population over the year. Copepods were sorted at 2˚C under a stereo microscope for stage (CV) using morphological characteristics. For each time point, 3 replicates of 25 *C*. *finmarchicus* CV were sorted (“Jan.1,” “Jan.2,” “Jan.3”; to “Dec.1,” “Dec.2,” “Dec.3”) (Figure 1). The choice to pool 25 individuals was made to: 1) get the sufficient amount of RNA required for RNA sequencing and 2) increase the number of individuals thereby increasing the representativeness of the population (75 copepods per time point, 900 copepods in total) and to decrease the effect of individual variability.

### 2.5 RNA extraction

Each replicate was transferred to a 2 ml Precellys^®^ homogenization tube (Bertin Instruments, France), containing a mix of 1.4 mm and 2.8 mm ceramic beads and homogenized in 600 µl of TRIzol^®^ reagent (ThermoFisher Scientific, USA) with a Precellys^®^ 24 Tissue Homogenizer (Bertin Instruments, France), using 2 × 15 s of homogenization at 1400 x *g* with a 10 s. break between. For RNA extraction, a Phenol/Chloroform‐based single‐step extraction in combination with a spin column based on solid phase extraction (Direct‐zol™ RNA MiniPrep Kit, Zymo Research, USA) was used. Genomic DNA was removed by DNase I digestion on column as part of the RNA extraction kit and total RNA was eluted in ultra‐pure water. Samples were sent to GeT‐PlaGe core facility in dried‐ice for RNA sequencing.

### 2.6 RNA sequencing

RNA sequencing was performed at the GeT‐PlaGe core facility, INRAE Toulouse. The 36 RNA sequencing libraries were prepared according to Illumina's protocols using the Illumina TruSeq Stranded mRNA sample prep kit to analyze mRNA. Briefly, mRNAs were selected using poly‐T beads. Then, RNAs were fragmented to generate double‐stranded cDNA, and adaptors were ligated to be sequenced. Eleven cycles of PCR were applied to amplify libraries. Library quality was assessed using a fragment analyzer (Advanced Analytical Technologies, Inc.), and libraries were quantified by qPCR using the Kapa Library Quantification Kit (Roche). RNA sequencing experiments were performed on a NovaSeq S4 lane (Illumina) using a paired‐end read length of 2 × 150 pb with the Illumina NovaSeq Reagent Kits. A total of 4,928,221,676 reads were sequenced. Raw RNA sequencing data are available as BioProject PRJNA734260 (Payton et al., 2022a).

### 2.7 Reads alignment and quantification

Thirty‐six RNA sequencing libraries (“Jan.1” to “Dec.3”) were obtained (Figure 1, Table 1, and Table S1). The number of reads per library was between 93 million and 191 million with an average of 137 ± 4 million (mean ±SE) reads (Table 2, Table S3). The RNA sequencing libraries’ read qualities were evaluated by checking the number of expected sequence, the GC percentage, the presence of adaptor, and the overexpressed sequences using FastQC (Andrews, 2010). Contamination was checked by aligning reads against *E*. *coli*, Yeast, and PhiX genomes. No further contamination investigations have been necessary considering the satisfactory reads alignment to the de novo reference transcriptome described thereafter and in the Section 3.

**TABLE 2:** Mapping results on the reference transcriptome (96,090 comps) before and after filtering. Details are available in Table S3

	Average number of aligned reads (million, mean ± SE)	Number of comps
Mapping results before filtering	102 ± 3	96,090
Mapping results after filtering	99 ± 3	35,357

The *C*. *finmarchicus* de novo transcriptome (Lenz et al., 2014), based on different life stages and deposited to Bioproject PRJNA236528, was used as the reference transcriptome. It is composed of 206,012 contigs comprising 96,090 unique components (“comps”) and presents good results of quality assessment, with a nearly complete BUSCO set (Lenz et al., 2014; Tarrant et al., 2019). To generate the quantification matrix, reads alignment to the reference transcriptome (Lenz et al., 2014) and quantification was performed with Salmon (Patro et al., 2017). An average of 102 ± 3 million reads (mean ± SE) were mapped to the 96,090 unique comps (Lenz et al., 2014) (Table 2, Table S3).

### 2.8 Filtering

The quantification matrix revealed one outlier component (comp92_c0_seq1; corresponding to the unique transcript GAXK01170082.1). The outlier comp was searched in the NCBI nucleotide collection (the collection consists of GenBank, EMBL, DDBJ, PDB, and RefSeq sequences) using basic local alignment search tool (BLAST) and was found to have 99.98% identity with a *C*. *finmarchicus* 18S ribosomal RNA gene (accession number MF993124.1). This result illustrates that despite the selection of mRNA using poly‐T beads, a small fraction of ribosomal RNA (rRNA) can remain and be variable between libraries (Abernathy & Overturf, 2016). 26.2% and 28.8% of mapped reads were aligned to this component in the samples “Mar.1” and “Jul.3,” respectively, against 0.4 ± 0.1% (mean ± SE) in all other samples, comprising the associated replicates “Mar.2,” “Mar.3,” “Jul.1,” and “Jul.2” (Table S3). For these reasons, and because of the clear increase in replicate correlations after filtering this outlier (see in Section 3), this component was removed. Additionally, the matrix was filtered with edgeR (Robinson et al., 2010) and only “comps” with more than 1 CPM (Count Per Million) in at least one sample were kept, providing a matrix of 35,357 unique “comps” and an average of 99 ± 3 mapped reads (mean ± SE) per sample (Table 2, Table S3). The quantification matrix for the 35,357 components after filtering is available in Figshare (Payton et al., 2022b).

### 2.9 Annotation

An annotation matrix was generated by combining two existing annotation works: Bioproject PRJNA628886 (Payton et al., 2020) and Bioproject PRJNA236528 (Lenz et al., 2014) (Table 3). Bioproject PRJNA628886 (Payton et al., 2020) consisted of the selection of the best annotation hit of *C*. *finmarchicus* reference transcriptome (Lenz et al., 2014) against NR, TREMBL, and Swissprot databases, using DIAMOND (Buchfink et al., 2015) (https://doi.org/10.6084/m9.figshare.c.5127704). Bioproject PRJNA236528 (Lenz et al., 2014) consisted of an annotation of the *C*. *finmarchicus* reference transcriptome (Lenz et al., 2014) against the non‐redundant (nr) protein database (https://doi.org/10.5061/dryad.11978). Details of the number of annotated components per databases are provided in Table 3. Joining the two annotation works led to 22,643 components over 35,357 with at least one annotation (Table 3). GO (Gene Ontology) annotation was done using Bioproject PRJNA628886 (Payton et al., 2020), in which InterProScan (Jones et al., 2014) provided annotations from many protein signature databases (https://doi.org/10.6084/m9.figshare.c.5127704). 15,726 components over 35,357 were assigned to a GO annotation (Table 3). The combined annotation matrix of the 35,357 components is available in Figshare (Payton et al., 2022b).

**TABLE 3:** Number (and percentage) of annotated comps in the working transcriptome (35,357 comps), using annotation works from Bioproject PRJNA628886 (Payton et al., 2020) and Bioproject PRJNA236528 (Lenz et al., 2014)

Dataset	Annotation source	Database	Annotated comps	Comps with at least one annotation
Working transcriptome (35,357 comps)	Bioproject PRJNA628886	NR	9779 (27.7%)	22,643 (64.0%)
		Trembl	7868 (22.3%)	
		SwissProt	3803 (10.8%)	
	Bioproject PRJNA236528	Non‐redundant database	21,938 (62.0%)	
	Bioproject PRJNA628886	InterProScan (GO)	15,726 (44.5%)	15,726 (44.5%)

### 2.10 Normalization

Non‐uniformities in sequencing depths or library sizes (the total number of mapped reads) between samples mean that the observed counts are not directly comparable between samples. A down‐sampling normalization was then proposed, constituting down‐sampling the mapped reads to the lowest number among the 36 samples (after filtering, Table S3), that is, to 66.7 million reads per sample for all samples, in order to adjust for differences in sequencing depth among samples. This normalization, previously shown suitable for rhythmic studies (Hughes et al., 2017; Koike et al., 2012; Li et al., 2015; Payton et al., 2021), is appropriate considering the high sequencing depth of the sample with the smaller library size (66.7 million reads after filtering, Table S3) (Li et al., 2015). This was performed with package R metaseqR (https://rdrr.io/bioc/metaseqR/man/downsample.counts.html). The quantification matrix of the 35,357 components after filtering and down‐sampling normalization is available in Figshare (Payton et al., 2022b).

### 2.11 Code availability

Parameters to software tools involved are described in the following paragraph.

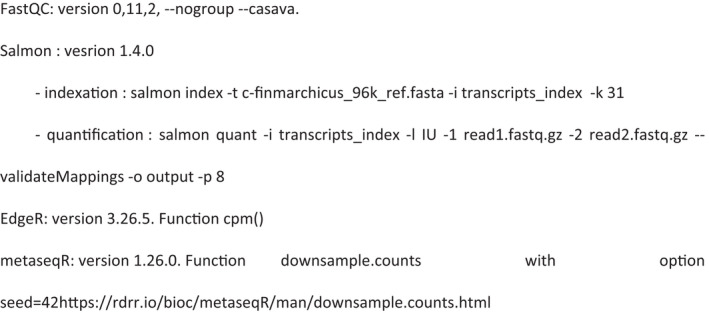

## 3 RESULTS

### 3.1 Species identification results

In all months no *C*. *helgolandicus*, only *C*. *finmarchicus*, were found between 140 and 50 m depth, where molecular sampling was performed. This result supports the assumption of extremely low risk of congener contamination.

### 3.2 Extraction and RNA integrity

RNA extraction procedures were performed with randomization of samples to ensure reliable and unbiased data production. RNA purity was assessed by OD measurements with a NanoDrop 8000 spectrophotometer (ThermoFisher Scientific, USA), and all 260/280 and 260/230 OD ratio was superior to 1.95. RNA integrity was evaluated with a Fragment Analyzer (Advanced Analytical Technologies, Inc., Iowa, USA; RNA Kit (15nt) Standard Sensitivity, Agilent). Due to a non‐conventional 28S/18S ribosomal ratio in this species, sample quality was evaluated on the electropherogram (Asai et al., 2015; DeLeo et al., 2018). No degradation in the inter region was observed. Total RNA samples were stored at −80°C.

### 3.3 Raw reads assessment and quantification overview

All samples passed the FastQC (Andrews, 2010) “base quality control.” No relevant contamination hit was found after the alignment against *E*. *coli*, Yeast, and PhiX. Mapping of the sequenced reads against the 96,090 unique “comps” of the reference transcriptome (Lenz et al., 2014) yielded an overall alignment of 102 ± 3 million reads (mean ± SE), that is, 74.5 ± 0.3% (mean ± SE) of total reads (Table 2, Table S3). This result is in accordance with the previously observed result on the de novo transcriptome of *C*. *finmarchicus* study (Lenz et al., 2014), where the alignment rate against unique comps was of 75%.

### 3.4 Effect of filtering and normalization on counts distribution

**FIGURE 2:**
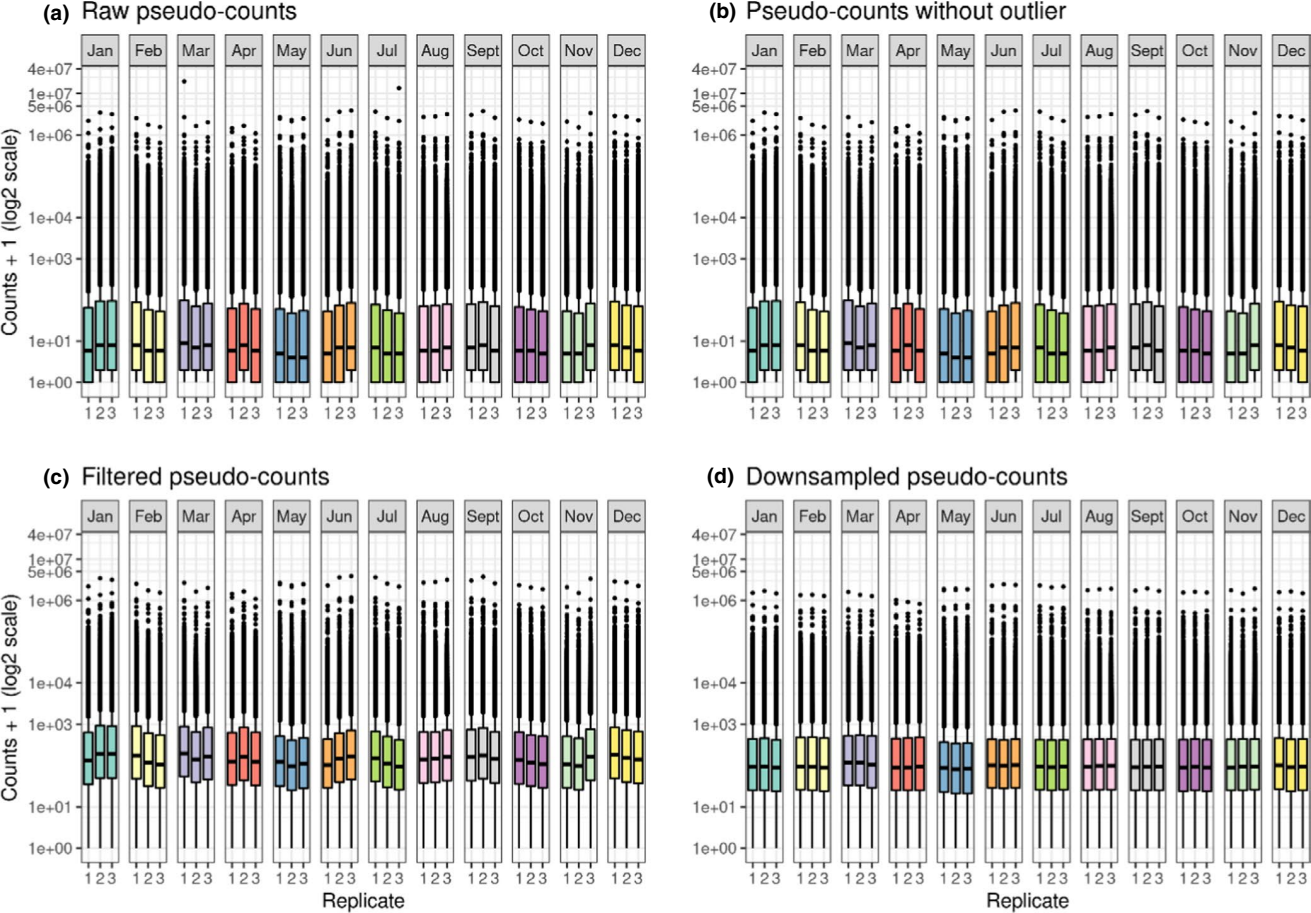
Counts distribution. Counts (expressed as pseudo‐counts, i.e. (counts +1) at the log_2_ scale) distribution: (a) before filtering the outlier component (96,090 “comps”), (b) after filtering the outlier component (96,089 “comps”), (c) after filtering components with less than 1 CPM in all samples (35,357 “comps”), and (d) after down‐sampling normalization (35,357 “comps”)

Samples counts distribution before and after removing the outlier component (comp92_c0_seq1; corresponding to the unique transcript GAXK01170082.1) clearly highlighted the overrepresentation of the latter in samples “Mar.1” and “Jul.3” (Figure 2a vs. Figure 2b). Moreover, filtering components expressed below 1 CPM in all samples clearly increased counts distribution (Figure 2b vs. Figure 2c). Finally, the down‐sampling normalization aligned the distribution of counts across samples, without observable impacts on the proportion of very low and very high expressed components (Figure 2c vs. Figure 2d) implying that this is a satisfactory approach.

### 3.5 Correlation between replicates

**FIGURE 3:**
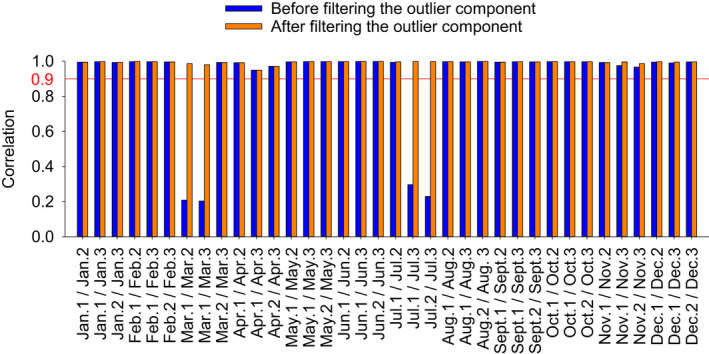
Correlation between replicates before and after filtering the outlier component (comp92_c0_seq1; GAXK01170082.1). Data are available in Table S4

Filtering the outlier component led to an increase in replicate correlations for March and July samples (Figure 3, Table S4). While correlations between “Mar. 1/Mar. 2,” “Mar. 1/Mar. 3” and “Jul. 1/Jul. 3,” “Jul. 2/Jul. 3” were of 0.21, 0.20, 0.30, and 0.23 before filtering; they increased to 0.99, 0.98, 1.00, and 1.00 respectively after filtering the outlier component, validating our methodology. Note that the minimal replicate correlation after filtering is 0.95 (“Apr. 1/Apr. 3”), revealing the good comparability of replicates in this study (Figure 3, Table S4).

### 3.6 Proportion of components expressed above the threshold of 1 CPM

Of the 96,090 components of the reference transcriptome (Lenz et al., 2014), 36.8% (35,357) were expressed above the threshold of 1 CPM (Table 2). This result corroborates previously observed results on the *C*. *finmarchicus* transcriptome (Lenz et al., 2014; Payton et al., 2020). Thus, a large proportion of the whole contigs (63.2%) exhibited an extremely low level of expression, representing only 0.39 ± 0.01% (mean ± SE) of total aligned reads per sample (Table S3).

## 4 CONCLUSION

This year‐round transcriptome dataset is of general interest for the understanding of annual oscillations of gene expression, specifically in organisms which perform diapause. Our study presents a significant contribution in providing the first in situ annual transcriptomic resource of a key zooplanktonic species, the copepod *C*. *finmarchicus*. This is significant since seasonal transcriptomic investigations in wild marine organisms are rare (Clark et al., 2013; Häfker et al., 2018; Lenz, Lieberman, et al., 2021; Lenz, Roncalli, et al., 2021; Roncalli et al., 2018, 2020, 2021; Semmouri et al., 2020; Tarrant et al., 2014). The proposed dataset is of interest and of high quality due to the unique and rigorous temporal resolution of sampling spanning the entire year and thereby facilitating rhythmic analysis (Thaben & Westermark, 2014). Furthermore, there is a high depth of the RNA sequencing with resulting high‐quality sequencing data. Indeed, the average sequencing depth of 137 ± 4 million reads (mean ±SE) per sample optimizes the investigation of gene expression in a species with a large genome (Bron et al., 2011; Choquet et al., 2019). We provide the raw reads, the quantification matrix after filtering, both before and after the normalization, and finally the combined annotation of the 35,357 components. Ultimately, this dataset will, among others, allow new insights into the understanding of mechanisms of animal diapause (Baumgartner & Tarrant, 2017; Hand et al., 2016), (circ)annual rhythms (Goto, 2013; Helm & Lincoln, 2017; Helm et al., 2013), and the consequences of climate‐driven environmental changes (Kharouba & Wolkovich, 2020).

## CONFLICT OF INTEREST

We have no conflict of interest.

## AUTHOR CONTRIBUTIONS

**Laura Payton:** Conceptualization (equal); Data curation (equal); Formal analysis (equal); Investigation (equal); Methodology (equal); Project administration (equal); Supervision (lead); Visualization (equal); Writing – original draft (lead); Writing – review & editing (equal). **Céline Noirot:** Data curation (lead); Formal analysis (equal); Investigation (equal); Methodology (equal); Software (equal); Visualization (equal); Writing – review & editing (equal). **Kim S. Last:** Conceptualization (equal); Data curation (equal); Funding acquisition (lead); Investigation (lead); Methodology (equal); Project administration (equal); Resources (equal); Supervision (lead); Writing – review & editing (equal). **Jordan Grigor:** Conceptualization (equal); Investigation (equal); Methodology (equal); Writing – review & editing (equal). **Lukas Hüppe:** Formal analysis (equal); Investigation (equal); Methodology (equal); Writing – review & editing (equal). **David V. P. Conway:** Formal analysis (equal); Investigation (equal); Methodology (equal); Validation (equal); Writing – review & editing (equal). **Mona Dannemeyer:** Formal analysis (equal); Methodology (equal); Writing – review & editing (equal). **Amandine Suin:** Investigation (equal); Methodology (equal); Writing – review & editing (equal). **Bettina Meyer:** Conceptualization (equal); Funding acquisition (lead); Methodology (equal); Project administration (equal); Supervision (lead); Writing – review & editing (equal).

### Open Research Badges

This article has earned an Open Data Badge for making publicly available the digitally‐shareable data necessary to reproduce the reported results. The data is available at http://www.ncbi.nlm.nih.gov/bioproject/734260; https://doi.org/10.6084/m9.figshare.c.5450397.v1.

## Supporting information

Table S1‐S4

## Data Availability

Raw reads were gathered in the BioProject PRJNA734260 (Payton et al., 2022a; http://www.ncbi.nlm.nih.gov/bioproject/734260) which includes all BioSamples used for the study (detailed in Table S1). We also provide the following in Figshare (Payton et al., 2022b; https://doi.org/10.6084/m9.figshare.c.5450397.v1): the quantification matrix for the 35,357 components after filtering; the quantification matrix of the 35,357 components after filtering and down‐sampling normalization; the combined annotation of the 35,357 components. The publication complies with relevant national laws. We have no competing interests. Benefits generated from this research accrue from the sharing of our data and results on public databases as described above.
